# The Forensic Entomology Case Report—A Global Perspective

**DOI:** 10.3390/insects12040283

**Published:** 2021-03-25

**Authors:** Zanthé Kotzé, Sylvain Aimar, Jens Amendt, Gail S. Anderson, Luc Bourguignon, Martin J.R. Hall, Jeffery K. Tomberlin

**Affiliations:** 1 Department of Entomology, Texas A&M University, 400 Bizzell St., College Station, TX 77843, USA; jktomberlin@tamu.edu; 2 Forensics Fauna and Flora Unit, Forensic Sciences Laboratory of the French Gendarmerie, 95000 Pontoise, France; sylvain.aimar@gendarmerie.interieur.gouv.fr; 3 Institute of Legal Medicine, University Hospital Frankfurt/Main, Goethe-University, 60323 Frankfurt, Germany; amendt@em.uni-frankfurt.de; 4 Centre for Forensic Research, School of Criminology, Simon Fraser University, Burnaby, BC V5A 1S6, Canada; gail_anderson@sfu.ca; 5 National Institute for Criminalistics and Criminology, 1120 Brussels, Belgium; Luc.Bourguignon@just.fgov.be; 6 Department of Life Sciences, Natural History Museum, London SW7 5BD, UK; m.hall@nhm.ac.uk

**Keywords:** Calliphoridae, legislation, expert witness statement, criteria, limitations

## Abstract

**Simple Summary:**

Forensic entomologists are most often tasked with determining when arthropods colonized living or deceased vertebrates. In most cases, this estimation involves humans; however, pets, livestock, and other domesticated animals can also be illegally killed or victims of neglect. Globally, there is no standard format for the case report, and much of the content is based on the personal preferences of the analyst or standards set within a country. The article below proposes a general overview of sections to be considered when drafting a case report.

**Abstract:**

Forensic practitioners analyzing entomological evidence are faced with numerous challenges when presenting their findings to law practitioners, particularly in terms of terminology used to describe insect age, what this means for colonization time of remains, and the limitations to estimates made. Due to varying legal requirements in different countries, there is no standard format for the entomological case report prepared, nor any guidelines as to the sections that are required, optional or unnecessary in a case report. The authors herein propose sections that should be considered when drafting an entomological case report. The criteria under which entomological evidence is analyzed are discussed, as well as the limitations for each criterion. The concept of a global, standardized entomological case report is impossible to achieve due to national legislative differences, but the authors here propose a basic template which can be adapted and changed according to the needs of the practitioner. Furthermore, while the discussion is fairly detailed, capturing all differences between nations could not be accomplished, and those initiating casework for the first time are encouraged to engage other practicing forensic entomologists or professional associations within their own nation or region, to ensure a complete report is generated that meets lab or national requirements, prior to generating a finalized report.

## 1. Introduction

In the last ~20 years, developments in the field of forensic entomology have progressed greatly and at pace. These increased research efforts and applications have resulted in over 1100 research and review articles, and books, published since 2000 [1]. Despite the vast number of references available, one of the many challenges faced in allowing entomological evidence to be admitted into court is an understanding of what exactly forensic entomology entails, what information the arthropod evidence can provide, and the application of such information to the case at hand, in addition to meeting standards for a given legal system, such as the Daubert standard of admissibility in the USA [2] and ISO 17025 (predominantly in the European Union) [3]. Of course, such standards vary between legal systems. 

Due to such standards being in place in most parts of the world, efforts have been aimed at developing specific guidelines for forensic entomology. Accreditation standards, such as those implemented by the European Association for Forensic Entomology (EAFE) for the sampling and evaluation of entomological traces, and the certification of entomological experts by the American Board of Forensic Entomology (ABFE), as well as the accreditation of laboratories (such as the Forensics Sciences Laboratory of the French Gendarmerie and the laboratory of the Belgium Institut National de Criminalistique et Criminologie) [4], have allowed for entomological evidence to be admitted into courts and analyzed as part of the legal proceedings. Similar standards are being developed in the USA by the Organization of Scientific Area Committees for Forensic Science (OSAC) [5], -Crime Scene Investigation Subcommittee, Forensic Entomology Task Group (Tomberlin, personal communication).

Forensic entomologists are often tasked with determining “how long the victim has been dead” by law enforcement and other officials [6], and may provide a written report (or expert witness statement), detailing an estimated time frame since insects first colonized the remains, and also present expert testimony in Court. The purpose of this article is to provide guidance for the preparation of an entomological case report and clarify its interpretation, with an outline of criteria, terminology, limitations/restrictions and applications of medicolegal forensic entomology, for use by both students new to the field, as well as investigators and legal counsel for clarity in a court of law. This work may also aid seasoned practitioners to improve the presentation of their findings and pave the way for a universal minimum standard in entomological case reports worldwide, adapted to suit relevant legal circumstances. However, it should be noted that such recommendations will vary in terms of their applicability depending on the location and agency involved in the investigation.

## 2. Criteria and Limitations

The overarching principle of forensic entomology is based on the arrival of insects to remains shortly after death, whereafter eggs or larvae are deposited. Larvae develop on the remains, and the age of the oldest developmental stages can be determined when the remains are discovered [6]. Assuming no disturbance, and ideal conditions, the age of the oldest developmental stages is determined to be close to the interval since time of death, termed the minimum post-mortem interval (minPMI). However, there are a number of factors which can cause deviation from ideal conditions, and thus affect the age determination. The role of the author of a forensic entomology report is to identify the factors present in each case, evaluate their weight and influence, and explain their impact on the minPMI estimation.

Due to the ectothermic nature of insects, much of their physiology, ecology, and behavior has been documented in relation to environmental conditions, especially temperature, but also including humidity, light intensity and wind. Insects inhabiting carrion, which include mainly flies (Diptera: e.g., the families Calliphoridae, Fanniidae, Muscidae, Phoridae, Piophilidae, Sarcophagidae, Stratiomyidae, and Syrphidae) and beetles (Coleoptera: e.g., the families Carabidae, Cleridae, Dermestidae, Histeridae, Silphidae and Staphylinidae), have been extensively studied and various criteria have been set forth to evaluate their development and succession on remains for a forensic report. The author of a forensic report should never assume (i.e., accept as being true without proof) anything; nevertheless, there are certain criteria related to insect biology which have support from scientific study and can act as guides to an analysis of insect evidence, unless there is compelling evidence to the contrary. Some of the basic criteria used by forensic practitioners, as well as their limitations, are listed below [6]:

1. Environmental weather records in the area reflect those at the body, and thus directly affect the development of the arthropods present:
  The consideration of temperature is fundamental in estimating the age of insects [7,8]. The microclimates in which insects develop at a scene can potentially vary greatly from the temperatures provided by a nearby environmental monitor (e.g., national weather station). Regardless of the debate among scientists as to whether the temperatures to which the developing insects were exposed should be taken one-to-one from that monitor or modelled site-specifically, practitioners should clearly state which method of estimating temperature was used, e.g., whether it was nearby weather station data, scene temperature logger data, or some form of regression analysis based on these two sets of data. Challenges exist with each method, and there are numerous factors that may affect the developmental patterns within the parameters of the influence of temperature [9].
2. Published developmental datasets based on laboratory studies accurately reflect developmental patterns in the nature of the insect evidence collected:
  The vast majority of published and accepted insect developmental datasets have been derived under laboratory conditions. These conditions usually applied a range of temperature profiles (constant or with daily variations), controlled humidity and specified light: dark cycles. When developing in a natural environment, none of the above-mentioned factors are controlled, and can affect development accordingly [10,11,12]. Temperature cycles fluctuate greatly, both daily and seasonally [13], humidity is dependent on a number of factors, including season and precipitation, and light: dark cycles are highly dependent on season and region (not to mention possible artificial lighting conditions). Although some field studies have validated laboratory data [14], the general assumption that developmental patterns observed in the laboratory are reflected in natural environments may result in an under- or overestimate of larval developmental patterns. More validation studies between laboratory and field developmental data are needed, in pursuit of increasing the accuracy and precision of entomological estimates, as well as their reliability;
3. Colonization occurred after death (i.e., no myiasis):
  In certain situations, oviposition or larviposition may occur before death, for example, when the decedent has open and possibly necrotic wounds such as decubitus ulcers (bed sores). Myiasis is the colonization of a living vertebrate host by fly larvae [15], and if the victim is not discovered until after death, it may not be known whether the colonization occurred before or after death [16,17]. While this could lead to an overestimation of time since death if not considered, it could also provide new leads for the investigation, e.g., in cases of suspected neglect, where demonstration of ante-mortem myiasis can be crucial evidence [18].
4. Specimens collected and analyzed developed on the body of the victim:
  Contamination of insect evidence can occur from other organisms that are deceased and within close proximity of the remains under scrutiny. For example, in an outdoor case, empty puparia in soil samples from a crime scene could originate from flies that had developed on a dead animal in the immediate vicinity at an earlier time.
5. Carrion-colonizing Diptera are diurnal and do not usually oviposit at night:
  Nocturnal oviposition is very rare and is thus usually excluded from analyses. Historically, it was assumed that oviposition only occurs during the day and, thus, hours of darkness were not considered when estimating the minPMI based on calculating the time of oviposition [19,20,21].
6. Carrion-colonizing insects (specifically Diptera and Coleoptera) have free access to the body:
  Oviposition or larviposition on the deceased occurs shortly after death without hindrance (physical and/or temporal/seasonal). However, in medico-legal cases where entomological evidence is to be obtained, a decedent may be concealed in order to prevent law enforcement from finding the body. This concealment may include burial, wrapping or disposal in bodies of water. In such instances, carrion-colonizing arthropods are limited in their access to the remains, often only gaining access after the remains have been discovered or exposed by the elements or by scavengers. In such instances particularly, the entomological evidence obtained provides details regarding the period of environmental exposure, provided the remains have always been in the conditions of their discovery, but cannot provide more specific information regarding a time frame of the decedents’ death.
7. Faunal succession patterns on, in and under the body can be used in estimating colonization intervals:
  While faunal succession patterns are somewhat predictable [22], they are seasonally and environmentally-dependent, and depend largely on the faunal species present in an area [23,24]. However, precise estimates of exact species present and their arrival patterns at remains cannot be determined without conducting field trials in many different environments, and creating a database of these findings, which is an unrealistic task and not necessarily reproducible outside of an experimental framework. Producing an entomological estimate based solely on faunal succession patterns is not likely to be robust and will have large confidence intervals. In most cases, faunal data are presented in terms of overlapping time frames, from which a minPMI can then be estimated [25,26,27,28]. In some instances, species level data can be used to interpret successional data; however, such cases are rare [29].

While the above criteria and limitations are broadly applicable to most cases, it must be noted that each case containing entomological evidence is unique and should be analyzed accordingly—there is no “one size fits all” approach.

## 3. Use of Terminology

There have been some disputes in recent years regarding the terminology used by forensic practitioners concerning entomological evidence [30]. Historically, entomological evidence was used to estimate the postmortem interval [31,32]. This term implies that the time of death of the decedent could be accurately estimated using arthropods present but this does not consider that there may be a delay in colonization for many different reasons, e.g., in an enclosure without insect access, such as a car trunk or locked room. All such issues would affect access to the remains by arthropods. 

A myriad of alternative terms has been introduced to describe the activity of arthropods on remains. In some manner or another, each term that is used describes the time since arthropods have colonized the remains. The terms include: minPMI; post-colonization interval (PCI); and time of colonization (TOC) [33,34].

Irrespective of the terminology selected by the practitioner, it is critical that the reader understands what the term used in the report is referring to and what it means; namely, a period of time which has passed at least since the occurrence of death. The clarification of terms is important for interpretation of the report by individuals without entomological/scientific training, such as law practitioners or judges/jurors. 

## 4. Insect Identification and Reliability of Keys

Numerous dichotomous and pictographic keys exist for the identification of arthropods based on physical characteristics. These keys are still the most frequently used means of identification for both immature and adult specimens of forensic importance. In many instances, samples may be received by the practitioner that have been damaged or are missing body parts. In such situations, the use of a dichotomous or pictographic key may not be the best avenue, as these keys reference specific body regions. Resources such as Lucidcentral [35] allow for the identification of specimens that are missing aspects critical for identification, as the data are arranged in a spreadsheet rather than a dichotomous key format. Additionally, voucher specimens from museums may also be used for comparison and identification. 

With the advent of molecular identification techniques, such as DNA barcoding, arthropod identification, especially that of insect fragments, has become easier [36,37,38,39,40]. However, despite the vast number of researchers using databases such as GenBank, errors in gene sequences still exist, and are being continuously detected and corrected. One of the most important benefits of using techniques such as DNA for identification is the accurate differentiation of morphologically and behaviorally similar species (provided that the corresponding developmental data set are available), such as *Lucilia cuprina* (Wiedemann) and *L. sericata* (Meigen) [41], or *Hemilucilia segmentaria* (Fabricius) and *H. semidiaphana* (Rondani) (all Diptera: Calliphoridae) [37]. While these species are behaviorally and morphologically similar, they differ in their developmental patterns, so accurate identification is important to provide a reliable estimate of colonization periods [42].

Whichever method of identification is used by the practitioner to identify specimens must be mentioned in the report.

## 5. Recommended Sections and Explanations for an Entomological Case Report

The following proposed sections for an entomological case report have been adapted and extended from those proposed in the Standard Operating Protocol for medico-criminal case reports by the American Board of Forensic Entomology in 2009 (see Table 1 for template/summary).

1. Title indicating the contents of the report:
  - This should include a case number or legal system reference if applicable, as well as an indication that the report is of an entomological nature.

2. Analyst/practitioner contact information (including location):
  - This should include a working postal or email address and contact telephone number. The practitioner’s title and affiliation should be included.

3. Contact information of investigating officer or law practitioner (i.e., the person requesting the report):
  - Again, a working postal or email address and contact telephone number, plus title and affiliation of contact person included.

4. Instructions received:
  - This section should include a brief note on when and how the practitioner was contacted and a precise description of what was being asked of them by the investigating officer or other person requesting evidentiary analysis.

5. Case information (summary based on case file):
  - The purpose of this section is not to restate the entirety of the case file; rather, a brief summary of the biographic details of the case (date, time, location) and details pertinent to the victim(s) and entomological evidence.

6. Summary of insect evidence received, including at least a description of different developmental stages:
  - Many practitioners relabel evidence once received, based on their own preferences or the labeling system of their laboratory. Both the original evidence details and the renamed details should be included here, to cover the bases for chain of custody.
  - If vials containing evidence are split or repackaged for any reason, this should be indicated, with a reasonable explanation as to the reasoning behind repackaging (e.g., to change or add preservative). Vials that have been split into multiple sections must be relabeled, and new labels names indicated as well. This should follow chain of custody protocols as dictated by the regulating authority of the country.
  - For preserved evidence, the time of collection and time of preservation should be included.
  - If used, the preservation medium used by the practitioner should be indicated—often law enforcement officials do not have the necessary chemicals for preservation available at a collection scene and will use any suitable substance that is readily available (e.g., gin, vodka). Evidence is then analyzed and replaced into vials with a more standard ethanol preservative (the concentration of which must be indicated).
  - If live samples were provided, a detailed timeline of collection and transportation should be provided. This includes storage conditions (e.g., in coolers), if oxygen supply was limited in a sealed container, as well as dietary medium provided during transport. If samples were further reared once reaching the practitioner, rearing details (e.g., temperature, food supplied) should also be provided.
  - This would also be an appropriate section to indicate any external factors that may have affected insect colonization and development on the remains (such as concealment, found in a closed room/building with no open windows, thermostat on/off at constant temperature, as well as whether specimens had, at any point, faced refrigeration at a mortuary).

7. Environmental conditions obtained from weather stations:
  - Since weather stations are not always conveniently located near crime scenes, it is advisable to use the most relevant climatic data available, from a certified meteorological organization, such as the national meteorological institution of your jurisdiction/country, and also indicate if data loggers placed at the scene after body discovery have been used to reconstruct scene data.
  - The weather conditions at the time of insect collection should also be included if they were provided by the investigating officer.

8. Identification of species and biological background:
  - A brief background of the species identified should be presented, including geographic range and life cycle.
  - Suitable references that have been used in the analysis should also be included here. These should include references to the identification keys, voucher specimens and molecular techniques used for identification and comparison;
  - If a large number of specimens were provided, and only a subset analyzed, the criteria for subset selection should be mentioned.

9. Estimation of insect age:
  - This section, the bulk of the report, should be a brief summary of the estimation of the age of insect evidence based on temperature. This section should be broken down by species identified.

10. Case summary (including date range of colonization if applicable):
  - This section should highlight the most important findings and/or date range of colonization if applicable.
  - There are a number of ways this could be presented; it may be helpful to separate date ranges by species, with a conclusive statement encompassing the chosen range.

11. Criteria/caveats:
  - There is a long list of criteria as stated above. It should not be necessary to include all of these, but definitely those most pertinent to the specific case. These can also be included wherever relevant throughout the report, rather than as a separate section.

12. Declaration:
  - This should be based on the requirements of the legal system into which the report is submitted, which can vary greatly, and include a statement indicating that analyses were performed based on currently available information and, should more information become available, the findings are subject to change.

13. Signature:
  - The report should be signed in accordance with the local requirements for documents of legal value.

14. Accreditation statement (if available):
  - A list of professional qualifications of the author can be included here, including professional qualifications and the number of cases worked. This section may be omitted where national legislature does not require it, or where pre-accredited lists exist which include such information.

15. Reference list:
  - Citations identified in the report should be provided. These citations support the approaches, interpretation, and conclusions made in the report (see discussion below).

16. Supplementary documentation (if required):
  - Chain of custody documents (courier receipts etc.) if available.
  - Developmental data sets and calculations (upon request).
  - Tabulated weather data (upon request).

## 6. References and Citations Selected

References included in the case report should reflect the locality of the entomological evidence as far as possible, as well as being relevant to the species identified. Key aspects to consider when compiling/utilizing references include:

Locality:
  Where applicable, the datasets used should be based on insect populations as close to where the evidence was collected as possible. In cases where no local datasets are available, the practitioner should include a statement indicating that local data were not available. It must be noted, however, that developmental patterns by geographic separation may not always differ, and, in some cases, are comparable irrespective of location [34,43].
2. Species specificity:
  Where available, datasets pertaining to the actual species identified should be used. When this is not possible, it is preferable to exclude these species from analysis rather than use a dataset for a closely related species. However, the exclusion of such data is at the discretion of the practitioner, provided they maintain transparency regarding their findings, as sometimes it might be better to guide an investigation with a much more general conclusion based on data from a related species, suitably qualified with regard to accuracy, than to provide no conclusion. In such cases, the practitioner may refer to a generalized larval life cycle of the organisms concerned, or datasets for closely related species, to indicate a possible time of colonization estimate based on prevailing conditions. Irrespective of which sources a practitioner opts to utilize, these sources should be cited and the data should be accessible to any individual who is to read/analyze the submitted report.

## 7. Application and Conclusions 

The goal of any entomological report is a reliable estimate of the TOC, which some interpret as a minPMI, of vertebrate remains by arthropods in cases where such events occurred after death. The report should be written and constructed in such a way that it is understood by individuals regardless of their level of scientific training. The report should be grounded in scientific principles and expertise, but not so saturated with scientific jargon that non-scientists struggle to understand or interpret the report. We acknowledge that an entomological report is not a scientifically peer-reviewed paper, but it should be prepared to the same high standards and, in order to meet legal standards (such as the Daubert standard in the USA, and ISO 17025), a significant element of scientific expertise is required. Quality assurance in entomological reports is of the highest importance, and fact-based evidence will be critical. However, it is acknowledged that every report will also contain opinion-based evidence, based on the knowledge and experience of the person compiling the report, and the judge presiding over the case will need to acknowledge that each case is unique and will need to be considered, in some aspects, independently of other case reports and studies [4].

As indicated by the title of this communication, the points expressed above are recommended contents and points for consideration when compiling an entomological report for any legal purposes, including investigations of death, neglect, or stored product scenarios. As forensic practitioners, we understand that standards and legislation differ by country, and some sections may need to be revised to deviate slightly from those discussed, or the order of topics restructured to reflect the specific needs of the investigating officer or person requesting the report for its intended purpose (for example, not all entomological reports go to court). Without a global standard of legislature, the implementation of a standard entomological report may not be possible, but, at the very least, we hope this manuscript can provide a framework which entomological practitioners in any area can modify to develop a standardized report that is accepted by the respective jurisdiction in which the report is to be presented.

## Figures and Tables

**Table 1:** Proposed template for a forensic entomological report, with summary of content.

Proposed Report Section	Summary and/or Example of Content
**Title indicating the contents of the report**	e.g., “Estimation of post-mortem interval based on evaluation of entomological evidence.” File/case number as provided by law enforcement official, as well as location.
**Analyst/practitioner contact information (including location)**	Practitioner name, contact details (postal address, email address, telephone number), institutional affiliation.
**Requested by**	Officer/analyst/judge.
**Instructions received**	e.g., “Request for an estimate of post-mortem interval from entomological evidence collected from remains of (decedent/case number) on (date) at (location)”.
**Case information (summary based on case file)**	Person contacting practitioner, date contacted, evidence received, location of discovery, scene description (e.g., indoors, sealed room, thermostat reading (if applicable)).
**Summary of insect evidence received, e.g., including taxa, numbers received**	e.g., “500 larval specimens preserved in (preservation medium)—rough estimates can also be provided, e.g., 3 adult specimens (preserved), 25 live larval specimens on (rearing medium)”.
**Environmental conditions obtained from weather stations**	Local weather data for a relevant period—including an indication of where weather data were obtained from (e.g., national meteorological weather station, website, access date).
**Background of species identified and analysis of evidence**	Identification techniques, distribution of species, number of specimens analyzed, techniques used to age the insects based on their stage of development and environmental conditions.
**Estimation of insect age**	Estimation of age of insect evidence based on temperature, broken down by species.
**Case summary (including date range of colonization if applicable)**	If more than one species was identified, multiple date ranges should be presented with the broadest range of overlap presented.
**Criteria/caveats: these can be omitted as a separate section and included where they apply**	Caveats need to be stated when applicable to the case
**Declaration**	e.g., “Practitioner reserves the right to change the document as new information becomes available; report may only be reproduced in its entirety; the contents of the report are true and accurate”.
**Signature**	Full signature at the end of report.
**Accreditation statement (if available)**	e.g., “Member—American Board of Forensic Entomology”, as well as professional qualifications as applicable, and membership to local certification bodies.
Reference list (*)	Developmental datasets/voucher specimens used for comparison and identification.
Supplementary documentation (if required *)	Chain of custody documentation; summary of weather data; documented analysis (e.g., for insect aging methods); summary of entomological terms; professional qualifications.

* does not necessarily need to be provided with the report, but a statement of availability on request is then necessary.

## Data Availability

Not applicable.

## References

[1] Lei G., Liu F., Liu P., Zhou Y., Jiao T., Dang Y.-H. (2019). A bibliometric analysis of forensic entomology trends and perspectives worldwide over the last two decades (1998–2017). Forensic Sci. Int..

[2] Solomon S.M., Hackett E.J. (1996). Setting boundaries between science and law: Lessons from Daubert v. Merrell Dow Pharmaceuticals, Inc. Sci. Technol. Hum. Values.

[3] Rodima A., Vilbaste M., Saks O., Jakobson E., Koort E., Pihl V., Sooväli L., Jalukse L., Traks J., Virro K. (2005). ISO 17025 quality system in a university environment. Accredit. Qual. Assur..

[4] Amendt J. (2017). Forensic entomology. Forensic Sci. Res..

[5] NIST The Organization of Scientific Area Committees for Forensic Science. https://www.nist.gov/osac.

[6] Tarone A.M., Sanford M.R. (2017). Is PMI the hypothesis or the null hypothesis?. J. Med. Entomol..

[7] Ratte H.T., Hoffmann K.H. (1985). Temperature and insect development. Environmental Physiology and Biochemistry of Insects.

[8] Damos P., Savopoulou-Soultani M. (2012). Temperature-driven models for insect development and vital thermal requirements. Psyche.

[9] Lutz L., Amendt J. (2020). Stay cool or get hot? An applied primer for using temperature in forensic entomological case work. Sci. Justice.

[10] Bauer A.M., Bauer A., Tomberlin J.K. (2020). Effects of photoperiod on the development of forensically important blow fly *Chrysomya rufifacies* (Diptera: Calliphoridae). J. Med. Entomol..

[11] Bauer A., Bauer A.M., Tomberlin J.K. (2020). Impact of diet moisture on the development of the forensically important blow fly *Cochliomyia macellaria* (Fabricius) (Diptera: Calliphoridae). Forensic Sci. Int..

[12] Chen W., Yang L., Ren L., Shang Y., Wang S., Guo Y. (2019). Impact of constant versus fluctuating temperatures on the development and life history parameters of *Aldrichina grahami* (Diptera: Calliphoridae). Insects.

[13] Bernhardt J., Carleton A.M., LaMagna C. (2018). A comparison of daily temperature-averaging methods: Spatial variability and recent change for the CONUS. J. Clim..

[14] Donovan S.E., Hall M.J.R., Turner B.D., Moncrieff C.B. (2006). Larval growth rates of the blowfly, *Calliphora vicina*, over a range of temperatures. Med. Vet. Entomol..

[15] Zumpt F. (1965). Myiasis in Man and Animals in The Old World: A Textbook for Physicians, Veterinarians, and Zoologists.

[16] Goff M.L., Charbonneau S., Sullivan W. (1991). Presence of Fecal Material in Diapers as a Potential Source of Error in Estimations of Postmortem Interval Using Arthropod Development Rates. J. Forensic Sci..

[17] Vanin S., Bonizzoli M., Migliaccio M.L., Buoninsegni L.T., Bugelli V., Pinchi V., Focardi M. (2017). A case of insect colonization before the death. J. Forensic Sci..

[18] Hall M.J.R., Wall R.L., Stevens J.R. (2016). Traumatic Myiasis: A Neglected Disease in a Changing World. Annu. Rev. Entomol..

[19] Williams K.A., Wallman J.F., Lessard B.D., Kavazos C.R., Mazungula D.N., Villet M.H. (2017). Nocturnal oviposition behavior of blowflies (Diptera: Calliphoridae) in the southern hemisphere (South Africa and Australia) and its forensic implications. Forensic Sci. Med. Pathol..

[20] Amendt J., Zehner R., Reckel F. (2008). The nocturnal oviposition behaviour of blowflies (Diptera: Calliphoridae) in Central Europe and its forensic implications. Forensic Sci. Int..

[21] Greenberg B. (1990). Nocturnal oviposition behavior of blow flies (Diptera: Calliphoridae). J. Med. Entomol..

[22] Matuszewski S., Bajerlein D., Konwerski S., Szpila K. (2011). Insect succession and carrion decomposition in selected forests of Central Europe. Part 3: Succession of carrion fauna. Forensic Sci. Int..

[23] Wang J., Li Z., Chen Y., Chen Q., Yin X. (2008). The succession and development of insects on pig carcasses and their significances in estimating PMI in south China. Forensic Sci. Int..

[24] Schoenly K., Reid W. (1989). Dynamics of heterotrophic succession in carrion revisited. Oecologia.

[25] Anderson G.S., Van Laerhoven S.L. (1996). Initial studies on insect succession on carrion in southwestern British Columbia. J. Forensic Sci..

[26] Amendt J., Krettek R., Niess C., Zehner R., Bratzke H. (2000). Forensic entomology in Germany. Forensic Sci. Int..

[27] Wolff M., Uribe A., Ortiz A., Duque P. (2001). A preliminary study of forensic entomology in Medellín, Colombia. Forensic Sci. Int..

[28] Archer M.S. (2004). Annual variation in arrival and departure times of carrion insects at carcasses: Implications for succession studies in forensic entomology. Aust. J. Zool..

[29] Matuszewski S. (2011). Estimating the pre-appearance interval from temperature in *Necrodes littoralis* L.(Coleoptera: Silphidae). Forensic Sci. Int..

[30] Wells J.D. (2014). To the Editor: Misstatements Concerning Forensic Entomology Practice in Recent Publications. J. Med. Entomol..

[31] Nabity P.D., Higley L.G., Heng-moss T.M. (2006). Effects of temperature on development of *Phormia regina* (Diptera: Calliphoridae) and use of developmental data in determining time intervals in forensic entomology. J. Med. Entomol..

[32] Faucherre J., Cherix D., Wyss C. (1999). Behavior of *Calliphora vicina* (Diptera, Calliphoridae) under extreme conditions. J. Insect Behav..

[33] Tomberlin J.K., Mohr R., Benbow M.E., Tarone A.M., Van Laerhoven S. (2011). A roadmap for bridging basic and applied research in forensic entomology. Annu. Rev. Entomol..

[34] Amendt J., Campobasso C.P., Gaudry E., Reiter C., LeBlanc H.N., Hall M.J.R. (2007). Best practice in forensic entomology—Standards and guidelines. Int. J. Leg. Med..

[35] Lucid Central. https://www.lucidcentral.org/.

[36] Schroeder H., Klotzbach H., Elias S., Augustin C., Pueschel K. (2003). Use of PCR–RFLP for differentiation of calliphorid larvae (Diptera, Calliphoridae) on human corpses. Forensic Sci. Int..

[37] Thyssen P.J., Lessinger A.C., Azeredo-Espin A.M.L., Linhares A.X. (2005). The value of PCR-RFLP molecular markers for the differentiation of immature stages of two necrophagous flies (Diptera: Calliphoridae) of potential forensic importance. Neotrop. Entomol..

[38] Clarke L.J., Soubrier J., Weyrich L.S., Cooper A. (2014). Environmental metabarcodes for insects: In silico PCR reveals potential for taxonomic bias. Mol. Ecol. Resour..

[39] Bharti M., Singh B. (2017). DNA-based identification of forensically important blow flies (Diptera: Calliphoridae) from India. J. Med. Entomol..

[40] Yusseff-Vanegas S.Z., Agnarsson I. (2017). DNA-barcoding of forensically important blow flies (Diptera: Calliphoridae) in the Caribbean Region. PeerJ.

[41] Tourle R., Downie D., Villet M. (2009). Flies in the ointment: A morphological and molecular comparison of *Lucilia cuprina* and *Lucilia sericata* (Diptera: Calliphoridae) in South Africa. Med. Vet. Entomol..

[42] Sonet G., Jordaens K., Braet Y., Desmyter S. (2012). Why is the molecular identification of the forensically important blowfly species *Lucilia caesar* and *L. illustris* (family Calliphoridae) so problematic?. Forensic Sci. Int..

[43] Limsopatham K., Hall M.J.R., Zehner R., Zajac B.K., Verhoff M.A., Sontigun N., Sukontason K., Sukontason K.L., Amendt J. (2018). A molecular, morphological, and physiological comparison of English and German populations of *Calliphora vicina* (Diptera: Calliphoridae). PLoS ONE.

